# Vacancy formation in a 1D chain of dust particles in a DC discharge

**DOI:** 10.1038/s41598-024-62486-1

**Published:** 2024-06-10

**Authors:** A. V. Fedoseev, V. V. Litvinenko, E. V. Vasilieva, M. M. Vasiliev, O. F. Petrov

**Affiliations:** Joint Institute for High Temperatures RAS, Moscow, Russia 125412

**Keywords:** Plasma physics, Colloids, Rheology

## Abstract

The paper presents the first experimental observation of an atypical phenomena during self-organization of dust particles into a one-dimensional chain structure levitated vertically in the plasma of a DC glow discharge. Using a laser, the third (middle) dust particle was removed from the chain of five particles so that the positions of the remaining particles did not significantly change, and a vacancy occurred in the place of the removed particle. This state of the chain turned out to be very stable, which is confirmed by the observation of the subsequent exchange of places of the fourth and the fifth particles of the chain upon the action of the laser on the forth particle. After the exchange process, vertical positions of all particles (first, second, fourth and fifth) in the chain remained almost the same as before the exchange, and the vacancy at the position of the third particle was preserved. The experimental data and the video record of the observed phenomena as well as the estimates of the plasma parameters are presented. An assumption has been made about the mechanism of the discovered phenomena that at present discharge conditions both the vacancy formation and the dust particles positions exchange are possible due to a strong ion wakes which are formed behind the upstream dust particles of the chain.

## Introduction

The self-organization phenomena can be observed in nature, social, economic and many other processes, and researches of such processes are carried out in almost all areas of science. Self-organization is considered as an elementary process of evolution, leading to the formation of a more complex structure of the entire system^1–4^. It is inherent to open dissipative systems, which are far from thermodynamic equilibrium^1–4^. A decrease in entropy in such systems with sufficient dissipation of energy coming from outside and the establishment of entropy less than the equilibrium one leads to the formation of stationary ordered structures in them.

In contrast to ordinary passive Brownian particles, which are in thermal equilibrium with their surroundings, active Brownian particles are capable of absorbing energy from outside and converting it into their kinetic energy, which removes them from the thermodynamic equilibrium^5,6^. Thus, the systems of active Brownian particles can be considered as open systems far from thermodynamic equilibrium. In recent years, active Brownian motion has attracted great interest in various fields of science, such as biology, physics, sociology, materials science, etc.^5,6^. It is possible to study the processes of self-organization of micron-sized active Brownian particles into various strongly coupled structures in laboratory conditions if they are placed in a gas discharge plasma, where they are charged and captured by the electric field^7,8^. Another way to obtain structures of active Brownian particles is the levitation of diamagnetic particles in a non-uniform stationary magnetic field^9,10^. In both cases^7–10^, the kinetic motion of active Brownian particles can be easily induced by the action of laser radiation on them, and the state of the particles will be maintained due to free energy of radiation. For example, it was shown in^10^ that an increase in laser radiation on a cloud of particles causes self-organization and evolution of the particles system with transition to a more complex state with lower entropy, namely, the “chains” of particles are formed, and their number and length increases.

Another example of the self-organization of particles into ordered structures is the formation of linear “chains” of particles in electrorheological liquids, i.e. liquids with micron-sized polarizable particles suspended in them^11–13^. When an electric field is applied to such systems, the microparticles align along the field and thereby dramatically change the rheological properties of the liquid such as shear viscosity and dynamic modulus. This effect has wide potential for practical applications^14^. Electrorheological properties can also be exhibited by gas discharge plasma with micron-sized particles suspended in it^15–17^. When an external electric field is applied, the microparticles line up in so-called “strings” or “string-like clusters”^18–21^.

Self-organization of microparticles into one-dimensional, two-dimensional and three-dimensional ordered structures in low-temperature laboratory plasma is a well-known and very intriguing phenomenon^22–34^. When micron-sized solid particles are placed in the plasma of a radio frequency discharge or direct current discharge, they acquire a large negative charge, and a subsystem of these microparticles can become strongly coupled. The intense scattering of light by microscopic particles makes it possible to directly observe their movement and determine their coordinates and velocities. Therefore, the so-called “dusty plasma” can be used as a convenient tool for studying many different phenomena typical for non-ideal Coulomb systems including self-organization of dust particles into dust crystals^27–30^, phase transitions^35,36^, dust-acoustic waves^16,33,37^ and many others. Along with two-dimensional crystals^28–30^ and volumetric structures^18–21,31–34^ of dust particles, one-dimensional linear chains of dust particles were also obtained in laboratory gas-discharge plasma^22–26^. Various mechanisms have been proposed for the interaction of dust particles with each other and for the alignment of dust particles into linear chains^38–45^. Let us also mention large series of works on studying the formation of one-dimensional chains of dust particles in the Plasmakristall-4 (PK-4) facility under microgravity conditions^20,21,46–48^ and inside a glass box in an rf discharge^49–53^. It worth noting that a one-dimensional chain of dust particles is a simple and controllable object for studying the nature of self-organization of ordered structures, mechanisms of particle interaction, processes of momentum and energy transfer, phase transitions and wave propagation in them.

Despite more than thirty-year history of studying the formation of ordered plasma-dust structures, further theoretical and experimental studies are necessary to construct a complete picture of the phenomenon taking into account all valuable physical mechanisms. Over the entire period of research, various mechanisms that lead to the alignment of dust particles into the chains have been proposed. The simplest one that determines the structural parameters of a chain of dust particles, in particular, the distance between particles, is the balance of *compression* of the dust particles chain in the trapping electric potential and screened Coulomb *repulsion* between the charged dust particles. In addition, in most cases the chains of dust particles are formed along the vector of the external electric field. Due to the ion drift in the electric field^54^, positively charged ions are focused behind each negatively charged dust particle. As a result, the so-called “ion wake” is formed, i.e. a region with a predominant positive charge^55–60^. The presence of the ion wakes modifies the spatial distribution of the electric potential around the dust particles, and, consequently, the interaction potential of the neighboring particles. It is now generally accepted^55–60^ that the formation of ion wakes leads to increased interaction of dust particles and to their self-organization into quasi-one-dimensional (chain) dust structures. However, currently existing concepts and models of the formation of dust particles chains do not allow one to describe the phenomenon in which it is possible to remove one internal particle from the chain without changing the positions of the remaining particles. Such “a vacancy” in the chain should be vanished due to the chain compression by the external electric field.

Along with the question of creating ideal ordered structures, the need of creating such structures with stable vacancies in them arises. For example, it is known that the formation of vacancies in some materials significantly influence their mechanical, thermal, and electrical properties. Vacancies occur naturally in all crystalline materials^61^. In crystallography, a vacancy is a type of point defect in a crystal where an atom is missing. The presence of such defects changes the electrical and optical properties of the crystals. In semiconductors, vacancies play a crucial role varying electrical conductivity due to changes in electron flow caused by these vacancies. Another example of the use of variable presence or absence of an atom (vacancy) in a linear structure is the binary form of information storage, where 1 corresponds to the presence of an atom and 0 corresponds to a vacancy. Binary storage is an important concept in digital electronics, as all the digital systems operate using binary logic. Biological molecules such as RNA and DNA are also considered by some as data storage^62^. Thus, the vacancies play the role not only the “defects” but they are a crucial aspect of materials science, solid-state physics, information technology, biology, etc.

This paper presents the first experimental observations of the phenomenon of the formation of a “vacancy” in a one-dimensional chain of dust particles in a glow discharge plasma. The middle (third) particle from a chain of five particles was removed using a laser. The subsequent phenomenon of the exchange of the positions of the fourth and fifth particles is described, in which the positions of other particles remained practically unchanged as well as the vacancy in the place of the third particle was also preserved. Section "Experiment" presents a description of the experimental setup and the obtained experimental data. Section "Discussion" provides a discussion of the observed phenomena, including estimates of the main parameters of the discharge plasma and dust particles (Section "Estimations of plasma and dust particles parameters"), results of numerical calculation of the spatial distribution of the electric potential with an ion wake (Section "Self-consistent spatial distribution of the electric potential around a single dust particle") and a possible mechanism to explain the observed phenomena (Section "Possible mechanism of vacancy formation and particles positions exchange" ). The Conclusions are presented in Section "Conclusions".

## Experiment

**Figure 1:**
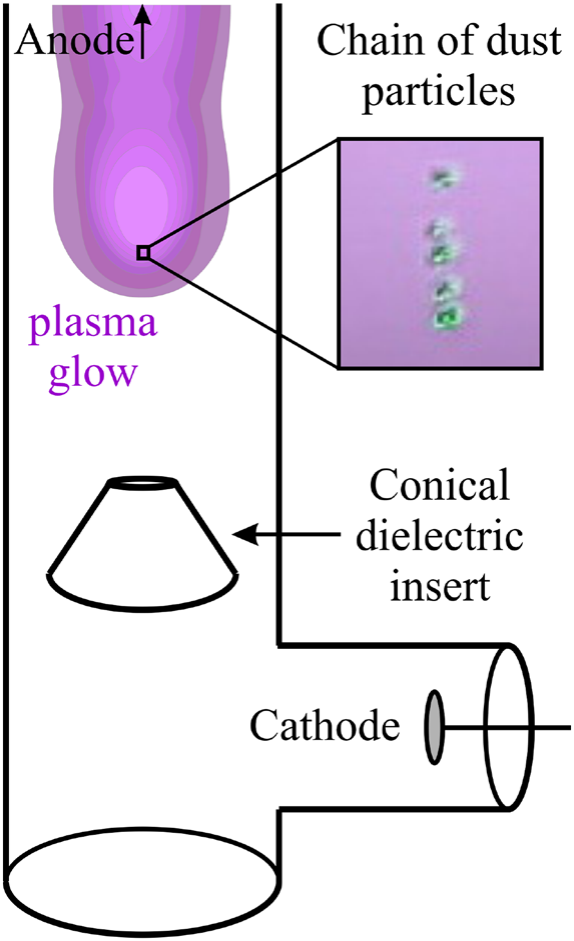
Scheme of the experimental setup: DC discharge tube, electrodes, conical dielectric insert, the plasma glow region above the insert and the position of the dust particles structure.

A direct current glow discharge plasma was generated in a vertically oriented glass discharge tube with an inner radius of *R*_*t*_ = 2 cm. Using a series-connected forevacuum and turbomolecular pumps, the air atmosphere was pumped out of the tube. After that, it was filled with an inert gas argon to the operating gas pressure *p* = 8 Pa. A cathode was placed in the lower side branch of the tube (see Fig. 1) and an anode was in the upper side branch. The distance between the cathode and the anode was 135 cm. When a voltage of *U*_*d*_ = 1650 V was applied between the electrodes, a positive column (PC) of a stratified glow discharge of direct current *I*_*d*_ = 0.8 mA was formed.

A container with dust particles was placed at the top of the tube. The injection of dust particles into the discharge was carried out through the mesh bottom of the container as a result of its mechanical vibration. Spherical monodisperse particles of melamine formaldehyde with density *ρ*_*d*_ = 1510 kg/m^3^ and radius *r*_*0*_ = 2.5 ± 0.1 µm were used as dust particles. Thus, the mass of one particle was approximately equal to *M*_*d*_ = 9.88 × 10^–14^ kg. A dust particle passing through the plasma of the discharge become charged as a result of the electron and ion flows onto its surface. As a result of the greater mobility of electrons, the particles were charged to large negative charge values (the estimates of the particle charge, *Q*_*d*_ ~ -10^4^ *e*, are presented below). Charged dust particles were captured by the electric field on the discharge tube axis in the lower part of the glow region of the striations, i.e. above the dielectric conical insert placed in the lower part of the discharge tube (see Fig. 1). For present gas discharge conditions, the formation of a stable chain structure of five dust particles was experimentally observed. To visualize the chain structure and dynamics of individual particles, they were illuminated with uniform scattered laser radiation with a power density of no more than 50 mW/cm^2^. The video recording was performed using a digital video camera placed in front of the chain structure. Subsequently, the records were processed and the positions of the particles at each moment in time were determined, thus, the trajectories of movement of each dust particle forming the chain were restored.

**Figure 2:**
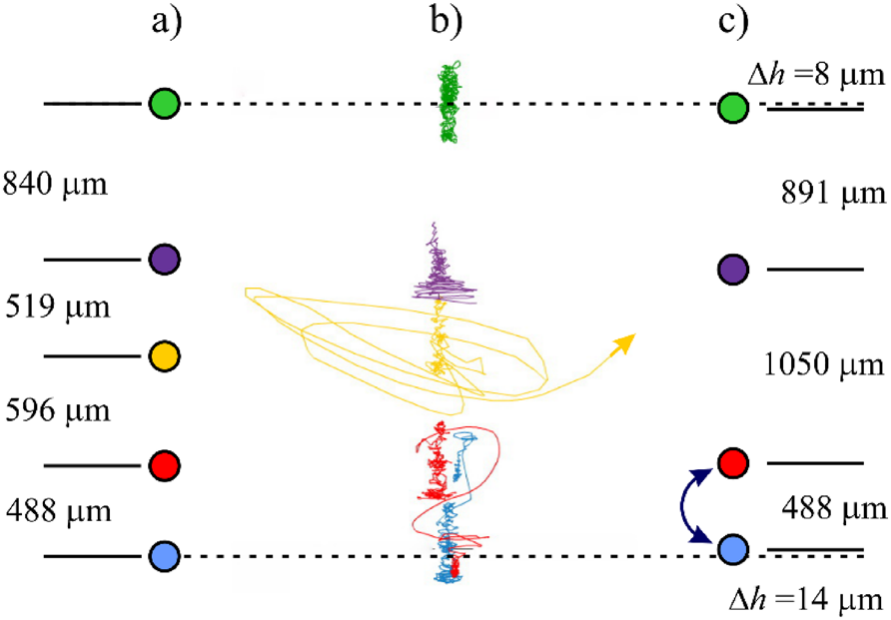
The positions of dust particles and the inter-particle distances: (**a**) in the chain of 5 particles before the removal of the central particle, and (**c**) in the chain of 4 particles after the removal of the central particle (vacancy is formed), (**b**) the trajectories of dust particles over a period of 10 s.

To study the process of formation of the chain-like structures of dust particles and the influence of the ion wakes on it, an experiment was carried out with the removal (“knocking out”) of the central particle from the chain of five particles (see Fig. 2 and Supplementary Video file). For this purpose, an additional source of the laser radiation was used. First, a low-power laser beam of the laser was focused on the central dust particle. Next, the laser radiation was blocked by a screen placed in front of the gas-discharge tube, and then the laser radiation power was increased to a maximum value (~ 1 W). After that, the screen was removed, and the powerful action of focused laser radiation “knocked out” the central particle from the chain.

Figure 2 schematically shows the dust particles vertical positions and the inter-particle distances before the particle removal (Fig. 2a) and after that (Fig. 2c). The trajectories of the particles movement during the abovementioned process lasting 10 s are presented in Fig. 2b. Different colors are used for different order number *k* of the particles in the chain. The top particle is denoted as the first (*k* = 1) particle of the chain. The trajectory of the third (central) particle movement under the action of the laser radiation is presented by yellow line. It is seen that after the beginning of the laser action, the particle made several circular movements around its position in the chain and finally it was “knocked out” from the chain (denoted by yellow arrow in Fig. 2b). Vertical positions and inter-particle distances right after the central particle removing are presented in Fig. 2c. It is very surprising that the positions of the remaining particles of the chain were not sufficiently change. The total length of the chain *L*_*b*_ = 2443 µm before the particle removal reduced to *L*_*a*_ = 2429 µm after that (1% change). It is seen that a vacancy in the place of the third particle of the chain was formed. The distance between the second (purple) and former fourth (red) particle decreased from 1115 to 1050 µm. It is worth noting that in most cases in other experiments, the neighboring particles approach each other at a distance approximately equal to the mean inter-particle distance between other particles of the chain. This is typically due to the balance of the chain compression by the electric field and the screened Coulomb repulsion between the particles with the charges of the same sign. The obtained state of the chain with the vacancy turned out to be stable in time. Moreover, in a couple of seconds later, the effect of the positions exchange of the fourth (red) and the fifth (blue) particles was observed, at which the vacancy in the chain was preserved. The trajectories of the lower two particles during their position exchange are clearly seen in Fig. 2b (also denoted by the dark blue double arrow in Fig. 2c). It should be noted that the chain of dust particles with formed vacancy is slightly drift in time in vertical direction up and down that is seen in Fig. 2b.

The above mentioned processes of the particle removal, vacancy formation and the subsequent particles positions exchange can be viewed in Supplementary Video file. The frame rate of the video is 150 frames per second. In it, vertical positions of the dust particles before the particle removal are indicated by red arrows, the action of the laser radiation on the central particle of the chain is indicated by long green arrow, new positions of the particles of the chain with vacancy are indicated by blue arrows, the process of particles positions exchange is surrounded by a pink circle.

**Figure 3:**
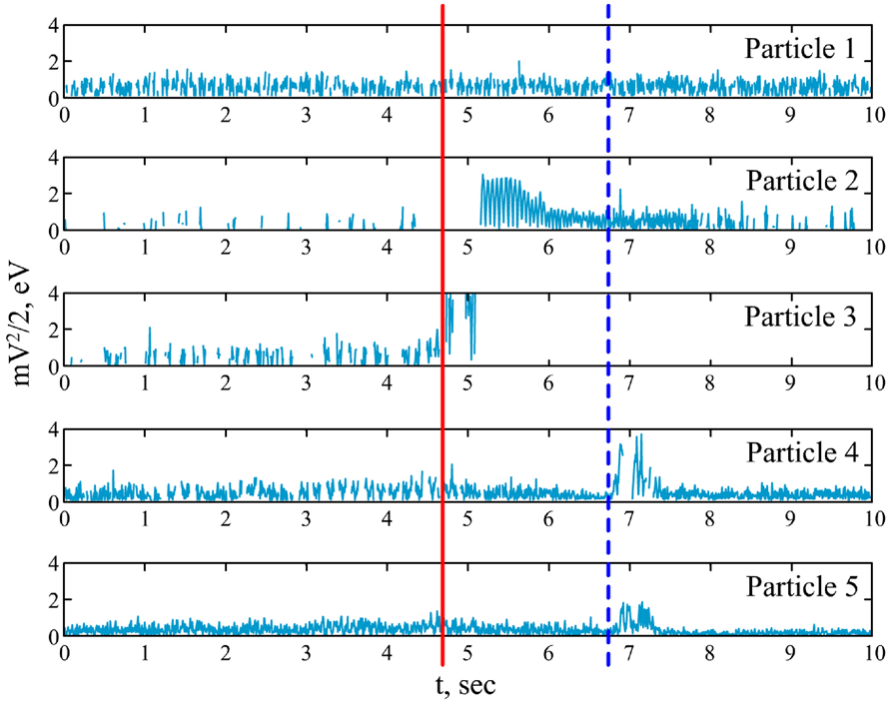
Time dependencies of the kinetic energy of the dust particles in the chain. Solid red line marks the starting moment of the laser action on the central particle. Dashed blue line marks the starting moment of the positions exchange of the fourth and the fifth particles.

In Fig. 3 the time dependencies of the kinetic energies of each dust particle in the chain are presented. It is seen that the energy of the first (top) particle of the chain remained almost unchanged that is in agreement with many experimental works on the chains of dust particles. After the beginning of the action of the laser radiation on the central (third) particle (vertical solid red line), the kinetic energy of the upper (second) particle was also increased by more than 5 times due to strong Coulomb coupling of the neighboring particles. After the removal of the third particle, its kinetic energy is denoted by zero, and the kinetic energy of the second particle gradually decreased. After that, the forth particle of the chain enters the action area of the laser radiation, and its kinetic energy raised several times (vertical dashed blue line). Once again, due to strong Coulomb coupling of neighbor particles, the kinetic energy of the neighboring fifth particle increased. At some moment, the exchange of the fourth and the fifth particles positions occurred that is also accompanied by increased energy of both particles.

## Discussion

To explain the phenomena of vacancy formation and the subsequent exchange of positions of two lower dust particles without changing the remaining structural properties of the chain, the main parameters of the discharge plasma and chain structure will be further estimated. For these parameters, using a model presented in previous works^43–45^, the self-consistent spatial distribution of the electric potential around a single dust particle will be calculated in order to estimate the properties of the ion wakes behind each dust particle of the chain. Based on the assessments of the main plasma-dust parameters and the calculated distribution of the electric potential, a possible mechanism of the observed phenomena will be proposed.

### Estimations of plasma and dust particles parameters

Let us estimate average value of the axial electric field strength < *E* > , in which the chain of dust particles is located. The total length of the discharge tube from the cathode to the anode is *L*_*t*_ = 135 cm. The voltage drop in the PC of the discharge, excluding the near-cathode voltage drop *U*_*c*_ ~ 400 V, remains *U*_*PC*_ = 1650—400 V = 1250 V. Hence, the average value of the electric field in the PC is < *E* >  = 9.25 V/cm. It should be noted that along the length of a stratum, the electric field strength can vary significantly^63–67^, i.e. at the head of the stratum it can reach 20–30 V/cm, and between the strata be a few volts per centimeter. For further analysis, the electric field strength in the region of dust particles is assumed to be equal to < *E* >  = 10 V/cm.

To estimate the parameters of the electrons of the discharge plasma, the BOLSIG + program was used^68^. BOLSIG + is a free software for the numerical solution of the Boltzmann kinetic equation for electrons in uniform electric field in collisional low-temperature plasmas. The electron energy distribution is determined by the balance between acceleration in the electric field and momentum and energy loss in collisions with neutral gas particles. The program permits to obtain the electron swarm parameters such as temperature, mobility and diffusion coefficients for the given value of the reduced electric field strength. For the reduced electric field < *E* > /*N*_*g*_ = 476 Td, where *N*_*g*_ = 0.21 × 10^16^ cm^-3^ is the density of neutral argon atoms, the program gives the following electron parameters: electron temperature *T*_*e*_ ≈ 6.43 eV and electron mobility *µ*_*e*_ ≈ 3.43 × 10^6^ cm^2^/(V s) for the specified gas density *N*_*g*_. Taking into account these values and the given discharge current *I*_*d*_, one can estimate the electron density *n*_*e*_ on the discharge axis. The electric current in the discharge tube *I*_*d*_ ≈ *e* < *n*_*e,r*_ > *S*_*t*_*µ*_*e*_ < *E*_*z*_ > is determined mainly by the electron drift, where *S*_*t*_ = π*R*_*t*_^2^ is the cross section of the tube. Thus, the cross-sectional average electron density in the tube is < *n*_*e*_(*r*) >  = 0.116 × 10^8^ cm^-3^. The electron density on the axis of the discharge tube *n*_*e*_(*r* = 0) is 2.32 times greater than the average < *n*_*e,r*_ > based on the assumption that *n*_*e*_(*r*) has a Bessel profile. It should be noted that the chain of particles is located above the silicone insert, at the exit of which a beam of electrons is focused. Hence, the density and the temperature of electrons in this region can be greater. Due to quasi-neutrality condition, the ion density is approximately equal to the electron density *n*_*i*_ ≈ *n*_*e*_* ≈ n*_*0*_, where *n*_*0*_ is the bulk plasma density. For further estimates, the plasma density in the region of the chain of dust particles is assumed to be equal to *n*_*0*_ ≈ 10^8^ cm^-3^.

Let us estimate the parameters of the ion component of the discharge plasma. It is assumed that the ion temperature is equal to *T*_*i*_ = *T*_*g*_ = 0.03 eV (348 K). Thus, the ratio of electron to ion temperatures is *τ* = *T*_*e*_/*T*_*i*_ = 215. The ion Debye length is equal to *λ*_*Di*_ = $\sqrt{\frac{k{T}_{i}}{4\pi {e}^{2}{n}_{i}}}$  = 129 μm, and the electron Debye length is *λ*_*De*_ = 1892 μm. The average ion mean free path is equal to *l*_*i*_ = 1/(*N*_*g*_2σ_*res*_) = 430 μm, where *σ*_*res*_ = 55.3 × 10^–16^ cm^2^ is the cross section of resonant collisions of argon ions with argon atoms. The drift velocity of Ar + ions in argon at < *E* > /*p* = 166.7 V/cm/Torr is *u*_*i*_ = 9·10^4^ cm/s according to^54^. The thermal speed of ions is $V_{Ti} = \sqrt {\frac{{kT_{i} }}{{m_{i} }}}$≈ 2.69·10^4^ cm/s, and the speed of sound (Bohm) is $c_{s} = \sqrt {\frac{{kT_{e} }}{{m_{i} }}}$≈ 40·10^4^ cm/s. Thus, the Mach number calculated relatively the ion thermal velocity turns out to be *M*_*T*_ ≈ 3, and the Mach number calculated relatively the speed of sound is *M*_*s*_ ≈ 0.2.

The estimations of the charge *Q*_*d*_ of dust particles are as follows. For the indicated parameters, the gravity force acting on a dust particle is equal to *F*_*g*_ = *M*_*d*_*g* = 0.96 × 10^–12^ N. Neglecting for simplicity the other forces acting on the particle (ion drag force, thermophoretic force, etc.), from the balance of gravitational and electrostatic forces, *F*_*E*_ = *Q*_*d*_*E* = -*eZ*_*d*_*E* = *F*_*g*_, it follows that the charge of the particle is equal to *Q*_*d*_ = -0.6 × 10^4^ *e*, and the charge number *Z*_*d*_ = 0.6 × 10^4^. An approximate estimate for collisionless plasma proposed in^58^, gives us the value of the charge number Z_*d*_ ≈ 1400 *r*_*0*_[µm] *T*_*e*_[eV] ≈ 2.25 × 10^4^. The accounting for the collisions of ions with gas atoms will lead to a decrease in the charge value. Besides, one can take into account the ion drag force in the balance of forces. The strength of the ion drag force *F*_*id*_ can be estimated using the formula presented in^69^. With the given parameters, it turns out that *F*_*id*_ ≈ 0.25 × 10^–12^ N. The ion drag force acts on the particle co-directional to the ions drift towards the lower electrode, i.e. in the same direction as gravity. From the balance of forces, *F*_*E*_ = *F*_*g*_ + *F*_*id*_, it follows that the charge of the particle is *Q*_*d*_ ≈ -0.76 × 10^4^ *e*. Summarizing above mentioned estimates, the charge of dust particle at present conditions is assumed to be about *Q*_*d*_ = -10^4^ *e*.

### Self-consistent spatial distribution of the electric potential around a single dust particle

In order to analyze the parameters of an ion wake produced behind a single dust particle in the ion flow induced by the external electric field, the self-consistent plasma parameters were calculated using numerical program presented in^43–45^. The calculations were carried out for the parameters of the experiment with vacancy formation, namely, the ratio of electron to ion temperatures *τ* = *T*_*e*_/*T*_*i*_ = 215, plasma density around the dust particles *n*_*0*_ = 10^8^ cm^-3^, ion Debye length *λ*_*Di*_ = 129 μm, and ion mean free path *l*_*i*_ = 430 μm.

The value of the electrostatic force *F*_*E*_, which is needed for the particle levitation against the gravitational force *F*_*g*_ and the ion drag force *F*_*id*_, depends on the charge *Q*_*d*_ of the dust particle and the external electric field strength *E*, *F*_*E*_ = *Q*_*d*_* E*. In turn, the charge of the dust particle, the plasma spatial distribution around the particle and the ion drag force depend on the external electric field strength. Thus, the numerical experiments were performed by variation of the external electric field strength. As a result, the equilibrium value of *E* is found at which the balance of all forces acting on the particle fulfilled, *F*_*d*_ = *F*_*g*_ + *F*_*id*_—*F*_*E*_ = 0. For the present conditions, the following self-consistently calculated equilibrium values of the dust particles and the plasma parameters were obtained: external electric field strength *E* ≈ 8.46 V/cm, charge of the dust particle *Q*_*d*_ ≈ -0.97 × 10^4^ *e*, ion drift velocity *u*_*i*_ ≈ 3.82 *V*_*Ti*_ ≈ 0.26 × *c*_*s*_ ≈ 10.28·10^4^ cm/s. The calculated parameters are in good agreement with the previously made estimates. Calculations also showed that the ion drag force is *F*_*id*_ ≈ 0.26 *F*_*E*_ ≈ 0.34 pN and the gravitational force is *F*_*g*_ = 0.74 *F*_*E*_ ≈ 0.96 pN, where *F*_*E*_ ≈ 1.3 pN and *F*_*g*_ + *F*_*id*_—*F*_*E*_ = 0.

**Figure 4:**
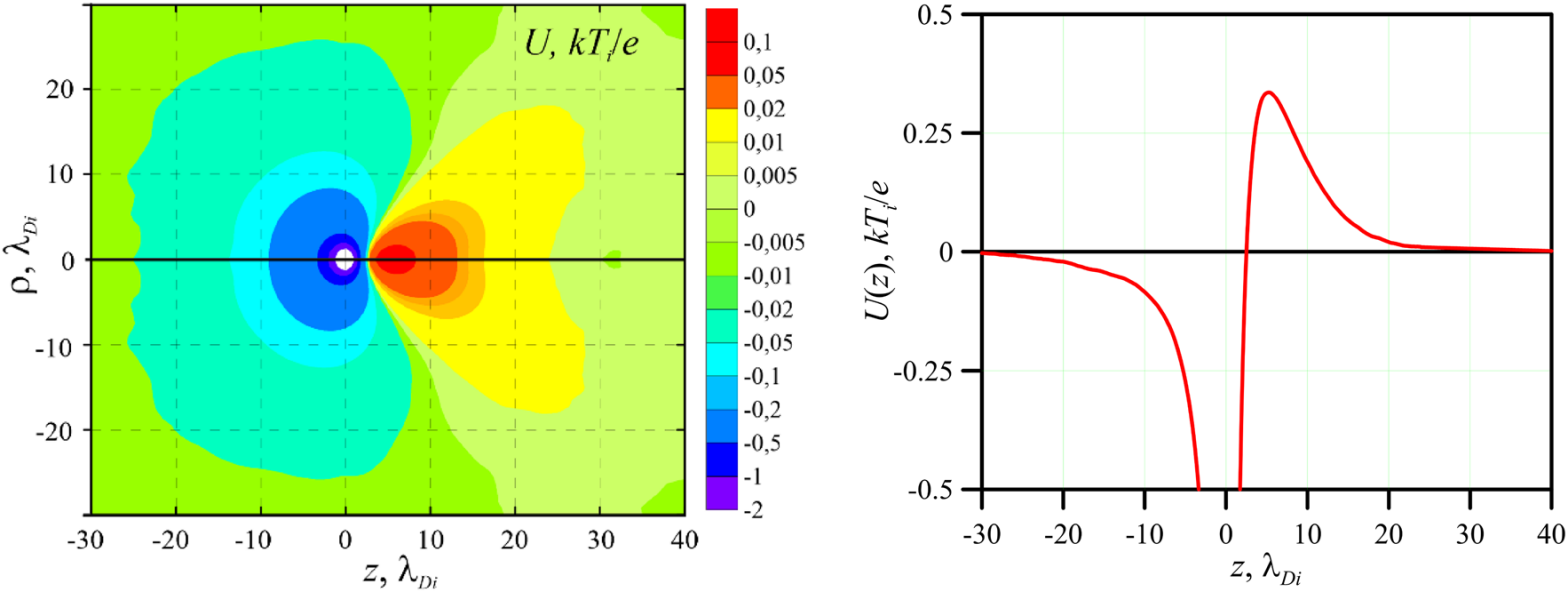
(**a**) Two-dimensional distribution *U*(*ρ*,*z*) and (**b**) axial distribution *U*(*z*) of the electric potential around a dust particle for the experimental parameters.

Figure 4a) presents two-dimensional distribution of the electric potential *U*(*ρ*,*z*) taking into account cylindrical symmetry of the problem, which is calculated for the equilibrium value of the external electric field *E* = 8.46 V/cm. Figure 4b) shows the spatial distribution of the electric potential *U*(*z*) along the *z* axis, passing through the center of the dust particle in direction of the vector of the external electric field ***E***. The presented potential *U*(*z*) is a superposition of the Coulomb potential of the dust particle itself and the electric potential of the space charge of the plasma (electrons and ions) surrounding the particle, which screens the particle potential. In the absence of the external electric field *E* = 0, the space charge of the plasma distributed around the particle is spherically isotropic, and the summarized electric potential of the charged particle and the ion cloud is described quite well by the screened Debye-Hückel potential. In the presence of the external electric field *E* ≠ 0, the ion cloud becomes anisotropic. As a result of the ion drift flow in the electric field towards the cathode, a so-called “ion wake” is formed, i.e. an area of focusing of positively charged ions behind a negatively charged particle. It can be seen that the pronounced ion wake with positive values of the electric potential is formed behind the dust particle in the region 2.5 < z < 30 *λ*_*Di*_ with maximum value of *U*_*1*_ ≈ 0.01 V at the distance *p*_*1*_ ≈ 5.3 *λ*_*Di*_ ≈ 683 µm from the particle.

### Possible mechanism of vacancy formation and particles positions exchange

It is known that in a DC discharge tube, the electric field strength *E*(*z*) increases down to the head of a stratum^63–66^. Thus, the entire chain of dust particles levitates in its equilibrium vertical position in the field of gravity^45,66^. As it was described in^45,66^, if the dust particles will be shifted out of the equilibrium position, for example, down to the head of the stratum, then they will come to the region with a larger value of the electric field, and, consequently, a larger electrostatic force will return it back to the equilibrium position. Similar reasoning can be carried out when the particles will be displaced up into the region with a lower electric field, as a result of which a resultant force will appear that will return it back to the equilibrium position.

Each dust particle of the chain is subjected to a number of forces which balance each other in the equilibrium. The main valuable forces are gravitational, electrostatic, ion drag and the Coulomb repulsive forces. Under certain conditions, due to ion focusing in a flowing plasma behind negatively charged dust particle, the pronounced ion wakes are produced, i.e. areas of the positive electric potential which are “attractors” for neighboring negatively charged dust particles. Such conditions include regimes with a large ratio of electron to ion temperatures, *τ* > 50, and with a high supersonic ion velocity, *u*_*i*_ > *V*_*Ti*_^55–60^. Above estimates showed that for the considered case of the formation of vacancies in chains of particles, the parameter *τ* = 215, and the Mach number calculated relatively to the thermal velocity of ions is *M*_*T*_ ~ 3. In addition, the presence of a pronounced ion wake behind a particle was also shown using numerical calculations in Section "Self-consistent spatial distribution of the electric potential around a single dust particle".

**Figure 5:**
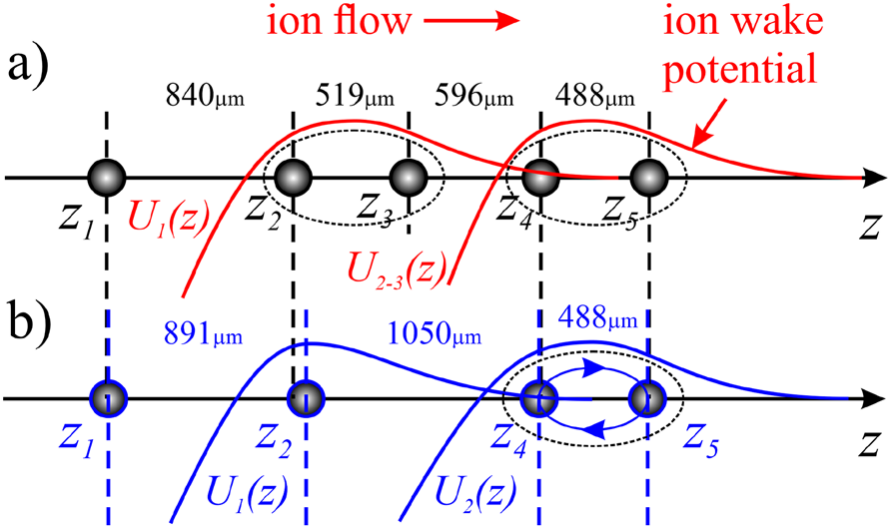
Equilibrium positions of dust particles in the chain taking into account pronounced ion wakes.

Figure 5a schematically shows a chain of five dust particles and their equilibrium spatial positions before the particle removal. The formation of ion wakes is taken into account. The experimental data shows that the pairs of particles “2–3” and “4–5” are at a closer distance to each other than other inter-particle distances (see Fig. 2). For example, the inter-particle distance between the second and the third dust particles *L*_*2-3*_ is sufficiently less than the distance between the first and the second ones *L*_*1-2*_, i.e. *L*_*2-3*_ ≈ 0.61 *L*_*1-2*_. The distance between the fourth and the fifth particles *L*_*4-5*_ is even less, *L*_*4-5*_ ≈ 0.58 *L*_*1-2*_. Moreover, it is clearly seen form the Supplementary Video file that the particles “2” and “3” have similar synchronous behavior, and the same thing happens with particles “4” and “5”. This gives us a reason to assume that before the formation of the vacancy, particles “2” and “3” as a pair are in the strong ion wake, which is formed behind the first particle, and the pair of particles “4–5” is in the joint ion wake behind pair “2–3” (see Fig. 5a).

The main assumption of the work is that after removal of the central particle from the chain, the pair of particles “4–5” remains in the strong ion wake of the second particle (see Fig. 5b). In this case, the total length of the entire chain of particles, i.e. distance *L*_*1-5*_ between the first and the fifth particles, decreased slightly. It is natural, since the screened Coulomb repulsion forces between all particles generally decreased. Despite the departure of the third particle, the fourth and the fifth particles remained approximately at the same vertical positions and at the same distance (488 µm) from each other (see Figs. 2 and 5). The calculation data presented in Section "Self-consistent spatial distribution of the electric potential around a single dust particle" showed that the distance from the particle to the maximum of the electric potential of the ion wake is about 683 μm. At the same time, the electric potential behind a particle has positive values at distances from 322 µm up to 3800 µm. This region with positive values of the electric potential plays a role of potential well for the negatively charged dust particles. Thus, after the vacancy formation, the pair of particles “4–5” lie entirely in the wake of the second particle, i.e. the fourth particle is at the distances *L*_*2-4*_ ≈ 1050 µm and the fifth particle is at the distances *L*_*2-5*_ ≈ 1538 µm from the second particle. It should be also stressed that the superposition of the ion focusing effects behind the first and the second dust particles could increase the ion wake behind the second particle.

From Fig. 2 and Supplementary Video file it is seen that after the formation of stable vacancy in the chain of dust particles, the chain as a whole spontaneously drifted up and down within a few seconds. Apparently, at some moment during the upward movement of the chain, the fourth particle accidentally fell into the region of the action of the high energy laser, which had previously knocked out the third particle. The laser action turned out to be indirect and insufficient to knock the fourth particle out of the chain, but the particle received a small transverse impulse, which was enough to exchange the positions with the fifth particle. It is well known^39,40^ that the electrostatic confinement of the negatively charged dust particles in the region of ion wake occurs not only in *z* direction (see Fig. 4b), but there is also a restoring force perpendicular to the ion flow. Thus, the 3D electrostatic potential well (see Fig. 4a) of strong ion wake behind the second particle captures the particles “4” and “5”, and the particles “4” and “5” exchanged their positions without leaving it. Thus, the fact of the formation of a stable vacancy in a chain of particles has been obtained, and the subsequent exchange of the positions of the fourth and the fifth particles confirms the proposed explanation of the vacancy formation consisting in the presence of the strong ion wake behind the second particle.

## Conclusions

The work presents clear experimental observations of the formation of a vacancy in a chain of dust particles and the subsequent exchange of positions of two particles without changing other structural parameters of the chain. To the best of the authors' knowledge, such phenomena have not yet been reported in the literature. Existing approaches and models of dust particles self-organization into ordered structures do not allow one to describe these effects.

The paper presents experimental data on the characteristics of the chain structure before and after the formation of the vacancy: inter-particle distances, trajectories of individual particles, their kinetic energy. The main parameters of the dust particles and the discharge plasma were estimated. Using a numerical model, the self-consistent spatial distribution of the electric potential around a single dust particle was calculated. It was shown that the observed phenomena occur in the strong ion wake regime. Based on the obtained data, the following assumption was made about the possible mechanism of the phenomena observed in the experiment. Under conditions of the formation of strong ion wakes in the chain of five dust particles, the second and the third particles as a pair are in the ion wake from the first dust particle, and a pair of particles four and five are in a joint ion wake from the particles two and three. After knocking out the third particle by a laser, a pair of particles four and five remains in the ion wake of the second particle, and subsequent exchange of their positions occurs without leaving it.

It should be noted that the numerical calculations presented in the paper were made for a single particle, the ion wakes behind a pair of particles have a complex structure, and the equilibrium positions of the particles both before and after the formation of the vacancy are determined by the superposition of many forces. To substantiate the proposed version of the vacancy formation in the dust particles chain and the subsequent particles positions exchange, further self-consistent numerical calculations for all five particles should be carried out.

The presented experimental data, estimations and numerical results can be useful in studying the nature of self-organization, in describing the formation of various ordered structures in strongly coupled Coulomb systems, including the formation of chains and crystals of dust particles in a low-temperature plasma. The experimental method of producing a vacancy in an ordered linear structure of dust particles in laboratory conditions can be an excellent example of changing the material properties which can be applied in the fields of solid-state physics, materials science, information technology, biology and others.

The Authors express their gratitude to intern D.B. Efimenko for his participation in the work of the team during the preparation and conduct of the experiment.

### Supplementary Information

Supplementary Video.

## Data Availability

The datasets used and/or analysed during the current study available from the corresponding author on reasonable request.
